# Genetic analysis of the orthologous *crt* and *mdr1* genes in *Plasmodium malariae* from Thailand and Myanmar

**DOI:** 10.1186/s12936-020-03391-6

**Published:** 2020-08-31

**Authors:** Yupawadee Pimpat, Naowarat Saralamba, Usa Boonyuen, Sasithon Pukrittayakamee, Francois Nosten, Frank Smithuis, Nicholas P. J. Day, Arjen M. Dondorp, Mallika Imwong

**Affiliations:** 1 Department of Molecular Tropical Medicine and Genetics, Faculty of Tropical Medicine, Mahidol University, Bangkok, Thailand; 2 Mahidol Oxford Tropical Medicine Research Unit, Faculty of Tropical Medicine, Mahidol University, Bangkok, Thailand; 3 Department of Clinical Tropical Medicine, Faculty of Tropical Medicine, Mahidol University, Bangkok, Thailand; 4 Shoklo Malaria Research Unit, Mahidol-Oxford Tropical Medicine Research Unit, Bangkok, Thailand; 5 Centre for Tropical Medicine, Nuffield Department of Clinical Medicine, University of Oxford, Oxford, UK; 6 Medical Action Myanmar, Yangon, Myanmar; 7 Centre for Tropical Medicine and Global Health, Churchill Hospital, University of Oxford, Oxford, UK

**Keywords:** Malaria, *Plasmodium malariae*, Chloroquine resistant transporter, Multidrug resistance proteins 1

## Abstract

**Background:**

*Plasmodium malariae* is a widely spread but neglected human malaria parasite, which causes chronic infections. Studies on genetic polymorphisms of anti-malarial drug target genes in *P. malariae* are limited. Previous reports have shown polymorphisms in the *P. malariae dihydrofolate reductase* gene associated with pyrimethamine resistance and linked to pyrimethamine drug pressure. This study investigated polymorphisms of the *P. malariae* homologous genes, *chloroquine resistant transporter* and *multidrug resistant 1*, associated with chloroquine and mefloquine resistance in *Plasmodium falciparum.*

**Methods:**

The orthologous *P. malariae crt* and *mdr1* genes were studied in 95 patients with *P. malariae* infection between 2002 and 2016 from Thailand (N = 51) and Myanmar (N = 44). Gene sequences were analysed using BioEdit, MEGA7, and DnaSP programs. Mutations and gene amplifications were compared with *P. falciparum* and *Plasmodium vivax* orthologous genes. Protein topology models derived from the observed *pmcrt* and *pmmdr1* haplotypes were constructed and analysed using Phyre2, SWISS MODEL and Discovery Studio Visualization V 17.2.

**Results:**

Two non-synonymous mutations were observed in exon 2 (H53P, 40%) and exon 8 (E278D, 44%) of *pmcrt*. The topology model indicated that H53P and E278D were located outside of the transmembrane domain and were unlikely to affect protein function. *Pmmdr1* was more diverse than *pmcrt*, with 10 non-synonymous and 3 synonymous mutations observed. Non-synonymous mutations were located in the parasite cytoplasmic site, transmembrane 11 and nucleotide binding domains 1 and 2. Polymorphisms conferring amino acid changes in the transmembrane and nucleotide binding domains were predicted to have some effect on PmMDR1 conformation, but were unlikely to affect protein function. All *P. malariae* parasites in this study contained a single copy of the *mdr1* gene.

**Conclusions:**

The observed polymorphisms in *pmcrt* and *pmmdr1* genes are unlikely to affect protein function and unlikely related to chloroquine drug pressure. Similarly, the absence of *pmmdr1* copy number variation suggests limited mefloquine drug pressure on the *P. malariae* parasite population, despite its long time use in Thailand for the treatment of falciparum malaria.

## Background

*Plasmodium malariae* is one of the five important human *Plasmodium* species, but its genome sequence has just been revealed [1, 2]. Recently, developed highly sensitive molecular diagnosis methods have shown that the prevalence of *P. malariae* in many malaria-endemic regions is higher than previously assumed [3]. There is limited information on the biology and molecular genetics of *P. malariae* as well as on anti-malarial drug resistance in this species for which long-term in vitro culture methods are lacking. Small scale in vitro drug susceptibility testing and clinical efficacy [4] showed that *P. malariae* in Thailand responds well to pyronaridine [5] and artesunate [6] but that fever clearance time was delayed after treatment with chloroquine [7]. However, *P. malariae* exhibits a 72-hour erythrocytic stage, which suggests that it may require a longer time to clear parasites after treatment. Therefore, the extended clearance time may not be an indicator for chloroquine resistance in *P. malariae* [8]. In vitro drug testing in *P. malariae* is further complicated by the low parasitaemias present in patients and the high proportion of mixed infections with other *Plasmodium* species. An alternative approach for monitoring anti-malarial drug resistance in *P. malariae* is the assessment of polymorphisms in anti-malarial drug target genes. Since the molecular markers for drug resistance are not well characterized in *P. malariae*, the orthologous gene markers associated with drug resistance in *Plasmodium falciparum* and *Plasmodium vivax* were considered in *P. malariae*. Earlier studies have reported polymorphisms in the *P. malariae dhps*, *dhfr* and *kelch* orthologues, which relate to sulfadoxine, pyrimethamine and artemisinin resistance, respectively.

*Plasmodium malariae* infection rate is generally underestimated and overlooked because it is often asymptomatic and mostly mixed with other *Plasmodium* species. Drug-resistant *P. falciparum* and *P. vivax* have been reported in many regions, including Thailand [9, 10] and Myanmar [11, 12]. Chloroquine-resistant *P. falciparum* was first documented in 1957 in Thailand which is now widely spread around the globe [13]. Polymorphisms of *pfcrt* and *pfmdr1* genes have been linked to chloroquine resistance with the key determinant of K76T mutation in *pfcrt* gene [10]. Several studies of *pfmdr1* supported that amplification and polymorphism were useful for prediction of chloroquine and mefloquine resistance [14–16]. The *pvmdr1* Y976F mutation has been found to correlate with reduced susceptibility to chloroquine [17, 18]. An increased copy number of *pvmdr1* has been found in Thailand [17, 18], suggesting that there is a relationship between *pvmdr1* copy number and mefloquine pressure. In the Greater Mekong Sub-region, previous drug pressure from chloroquine and mefloquine on the *P. malariae* parasite population is expected to be considerable, since these drugs have been used widely for the treatment of vivax and falciparum malaria, respectively. The current study focuses on the *P. malariae crt* and *mdr1* orthologue genes, which in *P. falciparum* are involved in chloroquine and mefloquine resistance. These genes were evaluated in *P. malariae* isolated of patients from Thailand and Myanmar between 2002 and 2016.

## Methods

### DNA extraction

Whole blood samples were collected from 95 *P. malariae*-infected patients from Thailand (51 samples) and Myanmar (44 samples) between 2002 and 2016 (Table 1). DNA was extracted using QIAamp DNA Mini Kit (Qiagen, Germany) and stored at −80 °C until use. *Plasmodium* species was confirmed by polymerase chain reaction based on *18 small*-*subunit ribosomal* RNA [19, 20].

**Table 1:** The samples used in the study

Country	Location	Year	Sample (N)	Total (N)
Thailand	Tak	2003–2008, 2012–2016	44	51
	Kanchanaburi	2002–2004	6	
	Cheangmai	2003	1	
Myanmar	Myanmar	2009	44	44
Total	95			

### Amplification of orthologous *crt* and *mdr1* genes from *Plasmodium malariae*

Specific primers for amplification of *pmcrt* and *pmmdr1* were designed based on the reference sequences (accession number LT594622.1 and LT594631.1). The primers and conditions used for amplification of *pmcrt* and *pmmdr1* are listed in Additional files 1 and 2. Semi-nested and nested PCR were carried out in 20 μl, 2 mM of MgCl_2_, 250 µM of dNTPs, 250 nM of both forward and reverse primers, 0.5U BIOTAQ DNA polymerase (Bioline, UK), and 2.0 μl of DNA template. The approximated concentration of DNA templates were 80 to 150 ng/μl. The primary PCR products were used as a template in the secondary PCR. Cycling conditions were: initial denaturation at 94 °C for 5 min then denaturation at 94 °C for 1 min, annealing for 1 min, and extension at 72 °C for 1 min plus 30 s, using 30 cycles during the first round and 35 cycles during the second round, follow by a final extension of 72 °C for 10 min. The positive PCR products were purified and submitted for DNA sequencing in South Korea (Macrogen Inc., Korea).

### DNA sequences analysis of *pmcrt* and *pmmdr1* genes

DNA sequences were analysed using Clustal software in the Bioedit package [21] together with reference sequences of *P. malariae* accession numbers LT594622.1 (*pmcrt*) and LT594631.1 (*pmmdr1*). DNA sequence polymorphisms and haplotypes patterns were analysed using DnaSP version 6.10.04 [22] and MEGA7 [23].

### Detection of *pmmdr1* copy number variation

To assess *pmmdr1* copy number variations, relative quantitative PCR was performed on the Applied Biosystems StepOnePlus™ (Applied Biosystems, USA). The primers for *pmmdr1* and *pmβ*–*tubulin* were *pmmdr1*F (5′- CAGATGTGGGAAACGACAATG-3′), *pmmdr1*R (5′-TAGAAGCTCCCTCCCCGTTT-3′), *pmβ*-*tubulin*F (5′-TGAAGCAACTGGAGGAAGGT-3′), and *pmβ*-*tubulin*R (5′-GGACCTGCTCGGACACTATC-3′). SsoFast™ EvaGreen^®^ Supermixes (Biorad, USA) was used as medium, and the thermal cycler profile was prepared according to the manufacturer’s instruction. As calibrator, a single copy control was constructed from a plasmid by insertion of *pmmdr1* (nucleotides [nt] 1102 to 1993) and *pmβ*–*tubulin* (nucleotides [nt] 1132 to 1215) fragments in a ratio of 1:1 into pGEM^®^-T Easy Vector (Promega, USA). In addition, the two-copy *pmmdr1* control was constructed by insertion of *pmmdr1* and *pmβ*–*tubulin* fragments in a ratio of 2:1 into the pGEM^®^-T Easy Vector (Promega, USA). Both of the control plasmids were confirmed their insertions by DNA sequencing. The *pmβ*–*tubulin* served as an internal control to calculate the relative amount of *pmmdr1* gene by comparing the C_t_ readings. Copy numbers were calculated as 2^−ΔΔCt^. All reactions were performed in triplicate. Samples with a copy number more than 1.5 were classified as multiple copies. Reactions were repeated at least twice in case the samples contained ΔΔC_t_ spread > 1.5, a C_t_ value > 35, or a *pmmdr*1 copy number higher than 1.3.

### Homology modelling of PmCRT and PmMDR1 proteins

The topology structure of PmCRT and PmMDR1 were predicted using Phyre2 [24] and SWISS-MODEL [25]. Homologous sequences of PmCRT and PmMDR1 proteins derived from different haplotypes were searched with PSI-Blast [26]. The secondary structure of PmCRT and PmMDR1 was predicted using Psi-pred 2.5 [27] and Disopred 2.4 softwares [28]. The membrane-spanning domains of PmCRT and PmMDR1 were predicted using Memsat_SVM program [29]. A multi-template approach was selected for prediction of the structural model of PmCRT and PmMDR1 proteins using SWISS-MODEL [25]. The model was evaluated with the VADAR tool and was visualized by Discovery Studio Visualizer V 17.2 [30].

## Results

### Genetic analysis of *pmcrt*

Purified PCR products covering the 14 exons of the *pmcrt* gene from all 95 *P. malariae* samples were sequenced and analysed. The assembled sequence covered the complete *pmcrt* coding sequence, with a size of 1272 nucleotides, encoding a protein of 423 amino acids. The multiple sequence alignments showed 9 nucleotide polymorphisms in exons 2, 8, 9, 10 and 12 at nucleotide positions A363C (40%), C346T (18.94%), C353T (53.68%), C394G (4.21%), A403T (28.42%), A2120T (44.2%), T2483T (2.10%), T2637A (1.05%), and A3151T (6.31%). There were 2 non-synonymous mutations in exons 2 and 8 at codons 53 (H53P) and 278 (E278D). The samples collected from Thailand showed more frequent mutations than samples collected from Myanmar. In Thailand, the prevalence of mutations in exons 2 (H53P) and 8 (E278D) were 66.66 and 60.78%, respectively, compared to Myanmar 9.09 and 25%, respectively (Table 2).

**Table 2:** Summary of *pmcrt* gene polymorphisms in Thailand and Myanmar

Prevalence of *Pmcrt* polymorphism											
Countries	Areas	N	Non-synonymous mutation		Synonymous mutation						
			Exon 2	Exon 8	Exon 2	Exon 2	Exon 2	Exon 2	Exon 9	Exon 10	Exon 12
			A363C CAT > CCT H53P	A2120T GAA > GAT E278D	C346T F47F	C353T L50L	C394G T63T	A403T I66I	T2483C N300N	T2637A L319L	A3151T I375I
Thailand	Kanchanaburi^a^	6	50% (3/6)	50% (3/6)	33% (2/6)	66.66% (4/6)	0% (0/6)	50% (3/6)	1.05% (1/6)	0% (0/6)	1.05% (1/6)
Thailand	Cheangmai^b^	1	0% (0/1)	100% (1/1)	0% (0/1)	0% (0/1)	0% (0/1)	0% (0/1)	0% (0/1)	0% (0/1)	0% (0/1)
Thailand	Tak^c^	44	70.45% (31/44)	61.36% (27/44)	31.81% (14/44)	61.36% (27/44)	0% (0/44)	47.72% (21/44)	0% (0/44)	0% (0/44)	9.09% (4/44)
Thailand	Total	51	66.66% (34/51)	60.78% (31/51)	31.37% (16/51)	60.78% (31/51)	0% (0/51)	47.05% (24/51)	1.96% (1/51)	0% (0/51)	9.80% (5/51)
Myanmar	Total^d^	44	9.09% (4/44)	25% (11/44)	4.54% (2/44)	45.45% (20/44)	9.09% (4/44)	6.81% (3/44)	2.27% (1/44)	2.27% (1/44)	2.27% (1/44)
Total in Thailand and Myanmar N = 95			40% (38/95)	44.21% (42/95)	18.94% (18/95)	53.68% (51/95)	4.21% (4/95)	28.42% (27/95)	2.10% (2/95)	1.05% (1/95)	6.31% (6/95)

Year of collection: ^a^2002–2004, ^b^2003, ^c^2003–2008 and 2012–2016, ^d^2009

Accession number of reference sequence: LT594622.1

Four haplotypes were characterized from 95 PmCRT sequences (Table 3). All 4 haplotypes were presented in samples from both Thailand and Myanmar. Haplotype 1 (H53P + E278D) was the most prevalent in Thailand (41.18%), whereas in Myanmar haplotype 4 (H53 + E278) was the most prevalent (68.18%). Haplotype 2 (H53E + E278) was observed in higher frequency in Thailand (25.49%, 13/51) than in Myanmar (6.82%, 3/44). There were ten samples (19.61% for Thailand and 22.73% for Myanmar) from both countries showing haplotype 3 (H53 + E278D).

**Table 3:** Haplotype patterns of PmCRT in Thailand and Myanmar

Haplotypes	H53P	E278D	Thailand % (N)	Myanmar % (N)	Frequency % (N)
1	P	D	41.18% (21/51)	2.27% (1/44)	23.15% (22/95)
2	P	E	25.49% (13/51)	6.82% (3/44)	0.16% (16/95)
3	H	D	19.61% (10/51)	22.73% (10/44)	21.05% (20/95)
4	H	E	13.73% (7/51)	68.18% (30/44)	38.94% (37/95)
Total			51	44	95

Accession number of reference sequence: LT594622.1

**Fig. 1:**
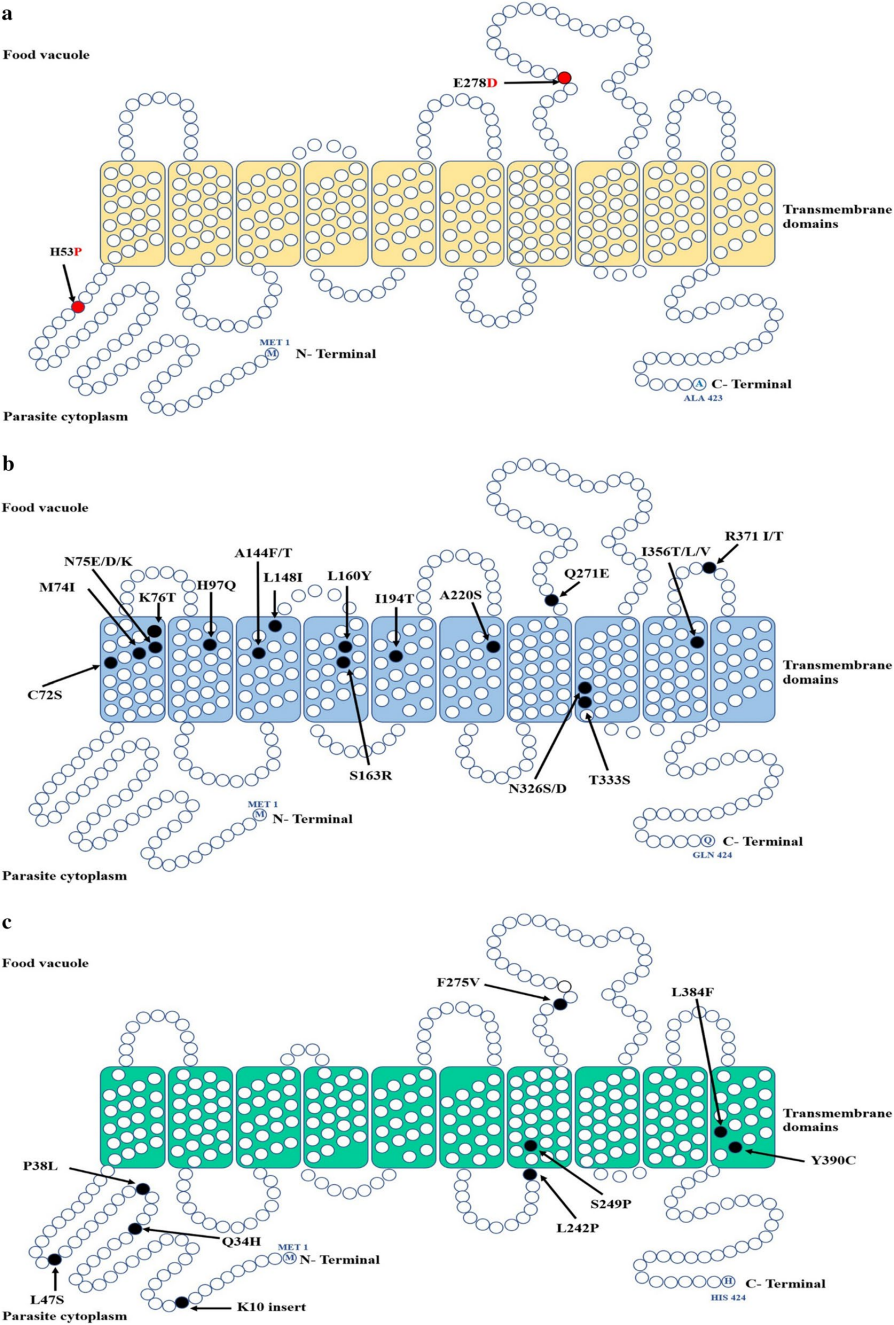
Topology model of CRT from different *Plasmodium* spp **a** PmCRT. **b**. PfCRT and **c** PvCRT. The mutated amino acid residues of PmCRT are highlighted in red circle. The previous reported mutatations found in PfCRT and PvCRT are labelled in black

For comparison, PmCRT haplotypes obtained from this study were aligned with the orthologous genes from human *Plasmodium* spp. The two-point mutations, H53P and E278D, found in *P. malariae* samples were not corresponded to the point mutations associated with chloroquine resistance described in *P. falciparum*. To predict the effect of these two-point mutations on PmCRT function, a topology model of PmCRT was constructed and then compared to PfCRT and PvCRT (Fig. 1). This showed that both H53P and E278D mutations are located outside the transmembrane domain (Fig. 1a), and are thus less likely to affect PmCRT function.

### Genetic analysis of *pmmdr1*

DNA sequence analysis of *P. malariae mdr1* gene (n = 95) revealed a single open reading frame of 4386 nucleotides, encoding a protein of 1461 amino acids. Results from multiple sequence alignments showed 13 single nucleotide polymorphisms. Of these, three were synonymous mutations at codons S77 (8.42%), S463 (42.10%), and L615 (31.57%). The most common polymorphism in *pmmdr1* was in codon 1389 (S463), prevalent of 39.21% in the samples collected from Thailand and of 45.45% in the samples from Myanmar. The identified 10 non-synonymous mutations and their prevalence included N6I (2.10%), Y7C (5.26%), L490I (4.21%), L1063F (16.84%), N1248I (5.26%), T1266S (12.63%), R1361S (3.15%), T1406S (4.21%), A1460S (4.21%), and A1460T (2.10%) (Table 4). For comparison, the non-synonymous mutations observed in *pmmdr1* gene were aligned to sequences of the orthologous *pfmdr1* and *pvmdr1* genes. This showed that the observed point mutations in *pmmdr1* were not corresponded to the *pfmdr1* or *pvmdr1* mutations associated with drug resistance (Additional file 3).

**Table 4:** Summary of *pmmdr*1 gene polymorphisms in Thailand and Myanmar

Prevalence of *pmmdr*1 polymorphism															
Countries	Areas	N	Non-synonymous mutation										Synonymous mutation		
			NT 17 AAC > ATC N6I	NT 20 TAT > TGT Y7C	NT 1468 CTT > ATT L490I	NT 3189 TTA > TTC L1063F	NT 3743 AAC > ATC N1248I	NT 3796 ACA > TCA T1266S	NT 4083 AGA > AGT R1361S	NT 4216 ACT > TCT T1406S	NT 4378 GCC > TCC A1460S	NT 4378 GCC > ACC A1460T	NT 231 TCC > TCT S77S	NT 1389 AGC > AGT S463S	NT 1845 TTG > TTA L615L
Thailand	Kanchanaburi^*a*^	6	0% (0/6)	0% (0/6)	0% (0/6)	66.66% (4/6)	0% (0/6)	0% (0/6)	1.05% (1/6)	1.05% (1/6)	0% (0/6)	0% (0/6)	0% (0/6)	83.33% (5/6)	33% (2/6)
	Cheangmai^b^	1	0% (0/1)	0% (0/1)	0% (0/1)	0% (0/1)	0% (0/1)	0% (0/1)	0% (0/1)	0% (0/1)	0% (0/1)	0% (0/1)	0% (0/1)	100% (1/1)	100% (1/1)
	Tak^c^	44	4.54% (2/44)	4.54% (2/44)	0% (0/44)	27.27% (12/44)	0% (0/44)	2.27% (1/44)	2.27% (1/44)	2.27% (1/44)	0% (0/44)	0% (0/44)	18.18% (8/44)	31.81% (14/44)	25% (11/44)
	Total	51	3.92% (2/51)	3.92% (2/51)	0% (0/51)	31.37% (16/51)	0% (0/51)	1.96% (1/51)	3.92% (2/51)	3.92% (2/51)	0% (0/51)	0% (0/51)	15.68% (8/51)	39.21% (20/51)	27.45% (14/51)
Myanmar	Total^d^	4	0% (0/44***)***	6.81% (3/44)	9.09% (4/44)	0% (0/44)	11.36% (5/44)	25% (11/44)	2.27% (1/44)	4.54% (2/44)	9.09% (4/44)	4.54% (2/44)	0% (0/44)	45.45% (20/44)	36.36% (16/44)
Total in Thailand and Myanmar N = 95			2.10% (2/95)	5.26% (5/95)	4.21% (4/95)	16.84% (16/95)	5.26% (5/95)	12.63% (12/95)	3.15% (3/95)	4.21% (4/95)	4.21% (4/95)	2.10% (2/95)	8.42% (8/95)	42.10% (40/95)	31.57% (30/95)

Year of collection: ^a^2002–2004, ^b^2003, ^c^2003–2008 and 2012–2016, ^d^2009

Accession number of reference sequence: LT594631.1

The PmMDR1 sequences showed 16 haplotypes patterns (Table 5). Samples from Myanmar were classified into 13 haplotypes while the samples collected from Thailand were classified into 6 haplotypes. Fifty-five samples, 30 from Thailand and 25 from Myanmar, were wild type (haplotype 16). Haplotypes 3 (NCLLNTRTAA) and 4 (NYLLNSRTAA) were found in both countries. A total of 10 haplotypes (haplotypes 5–7 and 9–15) were only identified in Myanmar, whereas three other haplotypes (haplotypes 1, 2, and 8), were identified only in Thailand (Table 5).

**Table 5:** Haplotype pattern of PmMDR1 in Thailand and Myanmar

Structure	Parasite cytoplasmic side		NBD1	TMD11	NBD2						Thailand % (N)	Myanmar % (N)	Frequency % (N)
Haplotypes	N6I	Y7C	L490I	L1063F	N1248I	T1266S	R1361S	T1406S	A1460S	A1460T			
1	N	Y	L	F	N	T	R	T	A	A	27.45% (14/51)	0% (0/44)	14.73% (14/95)
2	I	Y	L	L	N	T	R	T	A	A	3.92% (2/51)	0% (0/44)	2.10% (2/95)
3	N	C	L	L	N	T	R	T	A	A	3.92% (2/51)	2.27% (1/44)	3.57% (3/95)
4	N	Y	L	L	N	S	R	T	A	A	1.97% (1/51)	9.10% (4/44)	5.26% (5/95)
5	N	C	I	L	N	S	R	T	A	T	0% (0/51)	2.27% (1/44)	1.05% (1/95)
6	N	C	L	L	N	S	R	T	S	A	0% (0/51)	2.27% (1/44)	1.05% (1/95)
7	N	Y	L	L	N	S	R	T	S	A	0% (0/51)	2.27% (1/44)	1.05% (1/95)
8	N	Y	L	F	N	T	S	S	A	A	3.92% (2/51)	0% (0/44)	2.10% (2/95)
9	N	Y	I	L	N	T	R	T	A	A	0% (0/51)	6.82% (3/44)	3.57% (3/95)
10	N	Y	L	L	I	T	R	T	A	A	0% (0/51)	2.27% (1/44)	1.05% (1/95)
11	N	Y	L	L	I	S	R	T	A	A	0% (0/51)	6.82% (3/44)	3.57% (3/95)
12	N	Y	L	L	I	S	R	S	S	A	0% (0/51)	2.27% (1/44)	1.05% (1/95)
13	N	Y	L	L	N	T	R	S	S	A	0% (0/51)	2.27% (1/44)	1.05% (1/95)
14	N	Y	L	L	N	T	R	T	A	T	0% (0/51)	2.27% (1/44)	1.05% (1/95)
15	N	Y	L	L	N	T	S	T	A	A	0% (0/51)	2.27% (1/44)	1.05% (1/95)
16	N	Y	L	L	N	T	R	T	A	A	58.82% (30/51)	56.82% (25/44)	58.94% (55/95)
Total											51	44	100% (95)

Accession number of reference sequence: LT594631.1

**Fig. 2:**
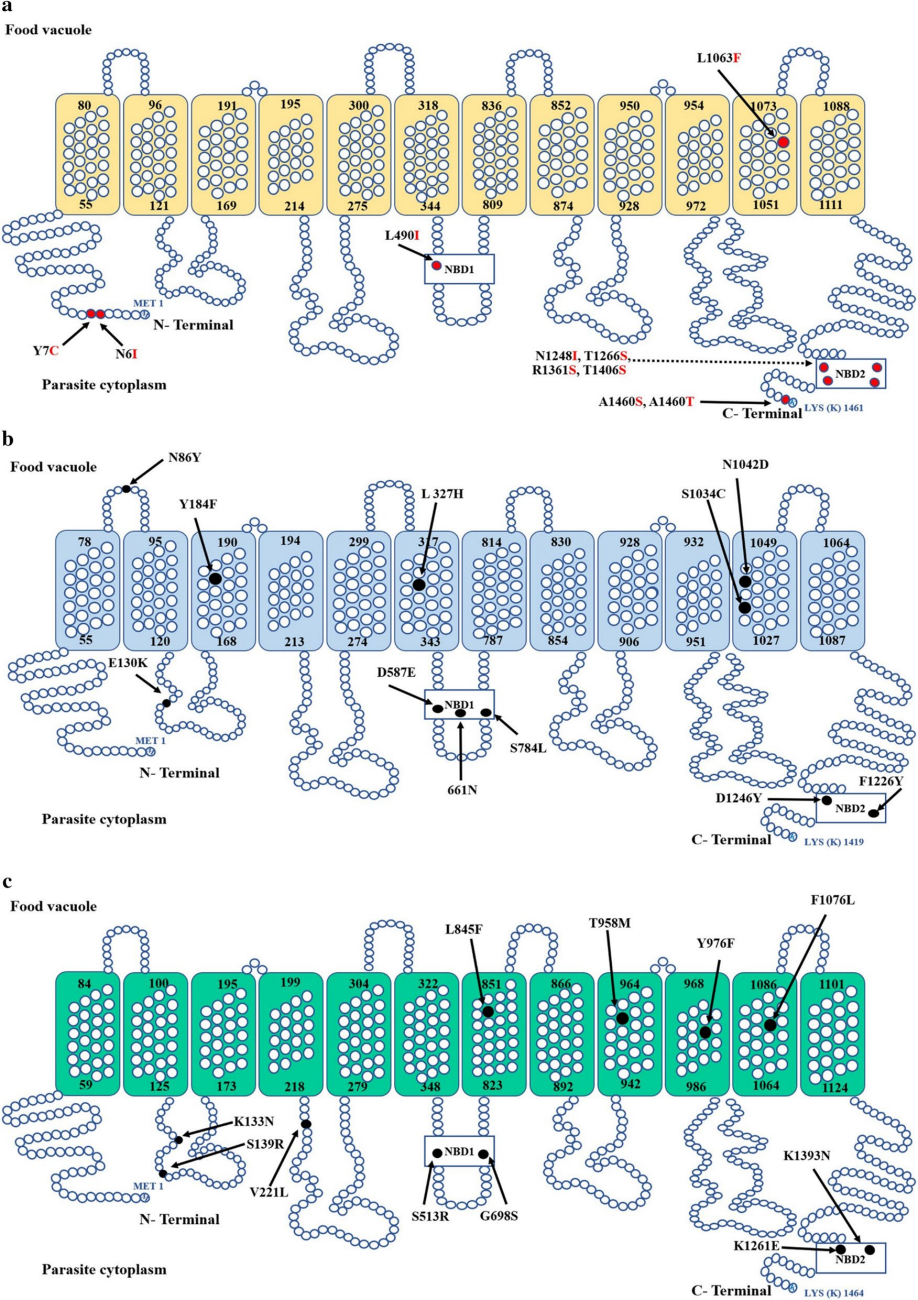
Topology model of MDR1 from different *Plasmodium* spp **a** PmMDR1, **b** PfMDR1 and **c** PvMDR1. The mutated amino acid residues of PmMDR1 are highlight in red circle. The previous reported mutation found in PfMDR1 and PvMDR1 are labelled in black

**Fig. 3:**
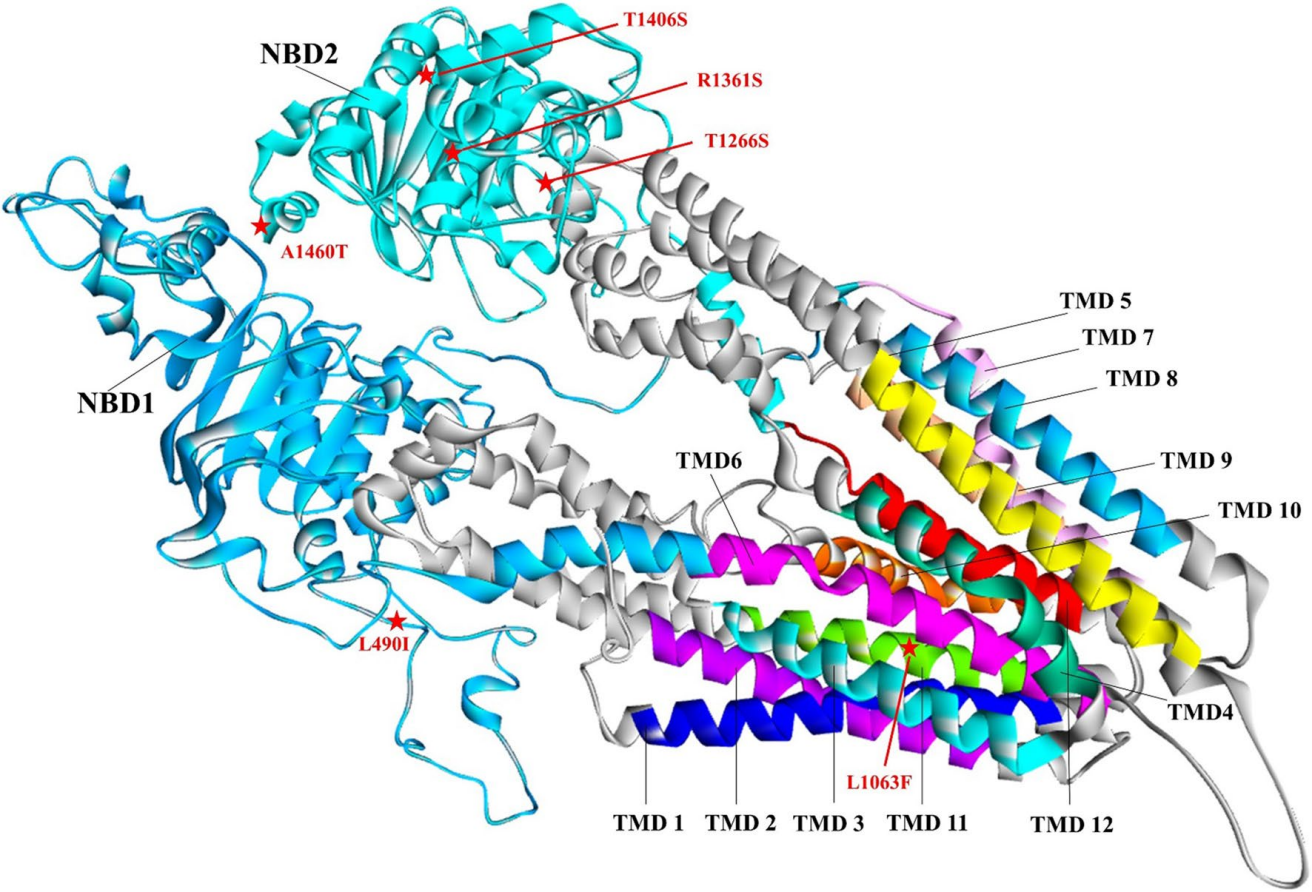
Three-dimensional structural model of PmMDR1 Transmembrane domains 1-12 are indicated as TMD1-TMD12. Two nucleotide binding domains are NBD1 and NBD2. Mutated residues in TMD11, NBD1, and NBD2 are labelled in red

A topology model of PmMDR1 was constructed and compared to the models of PfMDR1 and PvMDR1 (Fig. 2). PmMDR1 contains two nucleotide-binding domains (NBD1 and NBD2), facing the cytoplasm. Ten PmMDR1 substitution residues were identified: N6I, Y7C, L490I, L1063F, N1248I, T1266S, R1361S, T1406S, A1460S, and A1460T. Among the 10 mutations, N6I, Y7C were located on the cytoplasmic side, L490I located in nucleotide binding domain 1, L1063F located in transmembrane domain 11, N1248I, T1266S, R1361S, T1406S, A1460S, and A1460T located in NBD2. Although the mutations observed in *P. malariae* were not corresponding to residues associated with drug resistance reported previously in *P. falciparum*, the residues located in TMD11 and NBD might have an effect on PmMDR1 function. For this, structural models for each haplotype carrying mutations in TMD11 and NBD were constructed and analysed. The structural models representing these haplotypes showed similar characteristics and the mutated residues were predicted to have only a moderate effect on the PmMDR1 structure (Fig. 3).

Predicted changes in the physicochemical properties of each mutation were also addressed. For the L1063F mutant found in TMD11, leucine and phenylalanine share similar physicochemical properties, from which it can be inferred that this mutation has limited effect on the protein three-dimensional structure and function. A similar approach was followed for the four-point mutation identified in NBD, including L490I in NBD1 and T1266S, R1361S and A1460T/S in NBD2. To predict whether these point mutations affect the NBD structure and potentially its function, a structural model was constructed and residues involved in interactions between NBD and ATP were identified. For this, the highest conserved protein PGP1 from human was used as a template. Structural analysis of PmMDR1 model indicated that 20 and 19 residues are involved in ATP1 and ATP2 binding, respectively. Residue L490I was located outside of the ATP binding sites in NBD1. Residue T1266S, R1361S and A1460T/S in NBD2, were also located outside of the ATP binding sites.

**Fig. 4:**
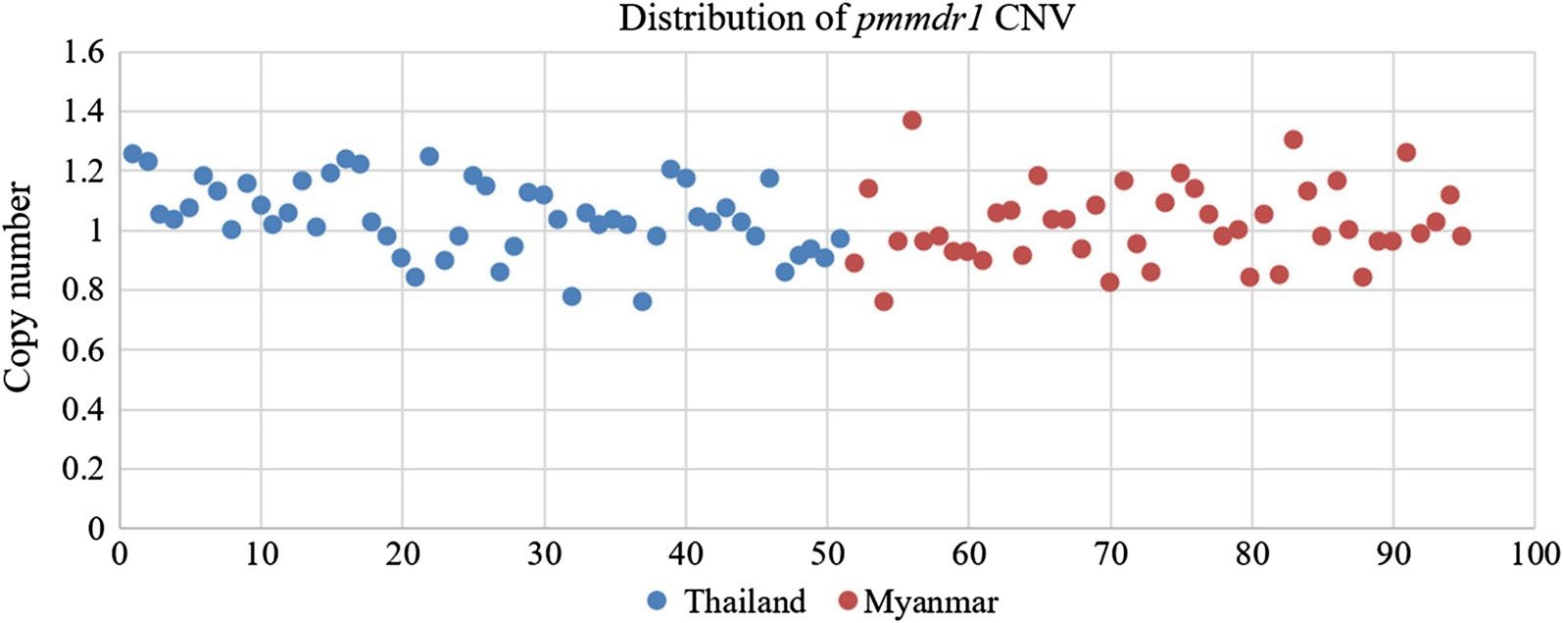
Distribution of *pmmdr1* copy number in Thailand and Myanmar

A total of 95 *P. malariae* isolates were assessed for *pmmdr1* gene amplification. The *pmmdr1* copy number in all samples ranged from 0.75 to 1.25 (Fig. 4), which represent a single copy of the *pmmdr1* gene.

## Discussion

In this study, the anti-malarial drug target genes, *pmcrt* and *pmmdr1*, were characterized in *P. malariae* samples collected from Thailand and Myanmar and were compared to the orthologous mutations in *pfcrt* and *pfmdr1* because polymorphisms in *P. falciparum* are well characterized for their association with chloroquine and mefloquine resistance [14–16, 31]. Since chloroquine and mefloquine have been used widely in the treatment of vivax and falciparum malaria in Thailand and Myanmar, it can be assumed that the *P. malariae* parasite population in these countries has also been exposed to these drugs, given the frequent co-infection with other human *Plasmodium* species in patients with *P. malariae* infection.

Nine nucleotide polymorphisms were identified in 5 out of the 14 exons of the *pmcrt* gene, with 2 non-synonymous mutations found in exon 2 (H53P) and exon 8 (E278D), both were not corresponding to the orthologous mutations in *P. falciparum* involved in chloroquine resistance. In *P. falciparum,* the majority of polymorphisms in PfCRT lay on exon 2, and mutations in PfCRT positions 72, 74, 75, and 76 are the main contributors to chloroquine resistance [32, 33]. The in silico topology model of PmCRT revealed that H53P and E278D are located in cytoplasmic region and food vacuole, which are outside of the transmembrane domain, and are thus considered unlikely to affect PmCRT function. Genetic polymorphisms in the *pmcrt* gene in both Thailand and Myanmar samples were limited, similar to previous studies describing very limited polymorphisms in the *pvcrt* gene in *P. vivax* populations in Thailand [34].

The number of polymorphisms in this study was higher in the *pmmdr1* gene. There were 13 SNPs, including 10 non-synonymous and 3 synonymous mutations, and none of these showed an equivalent position in *pfmdr1* associated with anti-malarial drug resistance in *P. falciparum*. Some of the *P. malariae* studied here were mixed infections with other species, which might have an impact on polymorphisms of *pmcrt* and *pmmdr1.* There were 12 and 11 mixed infections found in the samples from Thailand and Myanmar, respectively. The patterns of nucleotide polymorphisms in *pmcrt* and *pmmdr1* between single and mixed infections were compared. The proportion of mixed infected samples carrying mutations in Thailand and Myanmar was accounted for 14.29-22.72% (Additional file 4), suggesting that the mixed infections are unlikely to affect *pmcrt* and *pmmdr1* mutations in this study.

The mutations in *pmmdr1* codons L490I and L1063F correspond to the previously reported mutations in *P. vivax* at *pvmdr1* codons L493L and F1076L [17, 35]. In *P. vivax*, these polymorphisms have not been clearly linked to chloroquine resistance [36, 37]. The observed polymorphisms in *pmmdr1* were translated into a topology model of PmMDR1, showing that the mutations resulted in predicted protein changes located in the parasite cytoplasmic side of the protein, in nucleotide binding domains and one change in TMD11. In *P. falciparum*, residues in TMD11 were suggested as part of an anti-malarial binding pocket [38–40]. An in silico homology model of PmMDR1 was constructed and analysed to predict the effect of amino acid changes in TMD11 and NBD, which showed that the observed mutations were unlikely to affect the tertiary structure of the protein. Additionally, structural analysis of PmMDR1 polymorphisms found in NBD1 and NBD2 suggested that the mutations (L490I and L1063F) had only a moderate effect on the conformation of these domains, unlikely to affect the NBD function. Overall, the described polymorphisms were predicted to have insignificant impact on PmMDR1 protein morphology and function.

The samples used in this study were from Thailand and Myanmar collected at different periods of time, which may potentially affect the pattern of gene mutations [34]. The samples from Myanmar were collected in year 2009 in which artemether-lumefantrine was used as the first line treatment for uncomplicated malaria [41]. For Thailand, *P. malariae* were collected from two periods of time, year 2002–2008 and year 2012–2016. During those time periods, artesunate-mefloquine was used as the first-line treatment before it was changed to dihydroartemisinin-piperaquine in 2015 [41]. The *pmcrt* in Thailand showed more mutations (haplotypes 1-3, 81.48-91.67%) when compared to Myanmar (31.82%) (Additional file 5). There was no difference in mutation pattern of *pmmdr1* in both countries (Additional file 5). Although H53P and E278D mutations found in PmCRT were predicted that they were unlikely to have an impact on PmCRT function, the high proportion of those point mutations in Thailand might refer to geographical characteristics of the parasites. This will need to be confirmed in a larger sample size collected from different areas.

In *P. falciparum*, amplification of the *pfmdr1* gene is strongly associated with mefloquine resistance [14–16]. Despite the presumed long-term mefloquine drug pressure on the *P. malariae* parasite population in the study areas, none of *P. malariae* samples carried amplification of *pmmdr1.* Possible explanations include firstly, that *pmmdr1* amplification is not involved in mefloquine resistance in *P. malariae* and thus is not a good marker, and that there might be an alternative mechanism conferring mefloquine resistance in *P. malariae* other than *mdr1* amplification. Secondly, the parasite loads during infection of *P. malariae* is rarely exceed 1000 parasites per µl of blood. Thus, the number of parasites under selective pressure would be low for *P. malariae* and the likelihood of selecting resistant is lower. However, some of the potential drug resistance markers that have been studied in *P. malariae* might be under selective pressure such as *pmdhfr* [42, 43], *pmdhps* [44], and *pmkelch* [45]. Thirdly, although clinical *P. malariae* infection often presents as co-infection with other human *Plasmodium* species, there might be a large *P. malariae* reservoir outside of these patients with co-infection or a reservoir in non-human primates, so that overall *P. malariae* population has been limited to the selective pressure. The study of chloroquine-resistant *P. vivax* revealed that increased expression of *pvcrt* and *pvmdr1* are associated with chloroquine resistance [46, 47]. Moreover, the gene copy number of *pvcrt* was significantly higher in chloroquine-resistant *P. vivax* [48]. In addition to this study, expression level of *pmcrt* and *pmmdr1*, and the copy number variation of *pmcrt* should be evaluated.

## Conclusions

Polymorphisms in *pmmdr1* were more frequently observed than in *pmcrt*. The non-synonymous mutations found in both *pmcrt* and *pmmdr1* were unlikely to affect protein function. No amplification of *pmmdr1* was observed in this study. If the orthologous resistance genes in *P. malariae* are indeed associated with anti-malarial drug resistance in this *Plasmodium* species, the findings suggest limited chloroquine and mefloquine drug pressure on the *P. malariae* populations in the study regions. Alternatively, anti-malarial drug resistance in *P. malariae* could differ from that described in *P. falciparum* and *P. vivax*, which will require further investigation.

## Supplementary information

**Additional file 1.** The PCR primers and condition for amplification of *pmcrt* gene.

**Additional file 2.** The PCR primers and condition for amplification of *pmmdr1* gene.

**Additional file 3.** The equivalence position of *pmmdr1* gene compared with *pfmdr1* and *pvmdr1.*

**Additional file 4.** Summary of point mutations in *pmcrt* and *pmmdr1* and mixed *P. malariae* infections.

**Additional file 5.** Summary of point mutations in *pmcrt* and *pmmdr1* and sampling times.

## Data Availability

The nucleotide sequences of *pmcrt* and *pmmdr1* genes obtained from this study have been submitted to GenBank database under the Accession Numbers; MN623294-MN623342 (for *pmcrt*) and MN645870-MN645902 (for *pmmdr1*).

## Abbreviations

*pmcrt*: *Plasmodium malariae chloroquine resistant transporter*
*pfcrt*: *Plasmodium falciparum chloroquine resistant transporter*
*pvcrt*: *Plasmodium vivax chloroquine resistant transporter*
*pmmdr1*: *Plasmodium malariae multidrug resistance* 1
*pfmdr1*: *Plasmodium falciparum multidrug resistance* 1
*pvmdr1*: *Plasmodium vivax multidrug resistance* 1
SNPs: Single nucleotide polymorphisms
TMD: Transmembrane domain
NBD: Nucleotide binding domain
