# Supplementary material for: Genetic analysis of the orthologous crt and mdr1 genes in Plasmodium malariae from Thailand and Myanmar
Source: Malar J. 2020 Aug 31;19:315. doi: 10.1186/s12936-020-03391-6 (PMC7461347; doi:10.1186/s12936-020-03391-6)
Supplement: Supplementary file 1 — Additional file 1. The PCR primers and condition for amplification of pmcrt gene. [file 12936_2020_3391_MOESM1_ESM.docx]

**Additional file 1.** The PCR primers and condition for amplification of *pmcrt* gene

| **Fragment** | **Nested PCR** | **Name** | **Direction** | **Sequence (5'-3')** | **Annealing temperature (^o^C)** | **PCR product (bp)** |
| --- | --- | --- | --- | --- | --- | --- |
| F1 | 1^st^ | MCRT_F1_F1 | Forward | TATAAAACTACTGTGTAACAA |  |  |
|  |  | MCRT_F1_R1 | Reverse | ATGAAGCACTTCCGTTGGAT | 49 | 1254 |
|  | 2^st^ | MCRT_F1_F1 | Forward | TATAAAACTACTGTGTAACAA |  |  |
|  |  | MCRT_F1_R4 | Reverse | AAAACGGAACAAAGAAGGATAGAA | 49 | 252 |
|  | 2^st^ | MCRT_F1_F1 | Forward | TATAAAACTACTGTGTAACAA |  |  |
|  |  | MCRT_F1_R3 | Reverse | ATAGCCACATCCCCCTTTTC | 55 | 613 |
|  | 2^st^ | MCRT_F1_F1 | Forward | TATAAAACTACTGTGTAACAA |  |  |
|  |  | MCRT_F1_R2 | Reverse | TGTGCACATAACCCACACG | 50 | 949 |
|  | 2^st^ | MCRT_F1_F3 | Forward | TGTACGTGTGGGTTATGTGC |  |  |
|  |  | MCRT_F1_R1 | Reverse | ATGAAGCACTTCCGTTGGAT | 55 | 835 |
| F2 | 1^st^ | MCRT_F2_F2 | Forward | ACGGAAGTGCTTCATTGACC |  |  |
|  |  | MCRT_F2_R1 | Reverse | ACCAAGACCGCAATTCTACG | 52 | 1276 |
|  | 2^st^ | MCRT_F2_F2 | Forward | ACGGAAGTGCTTCATTGACC |  |  |
|  |  | MCRT_F2_R6 | Reverse | GGTTAGAAATAGCTAAAAGGAGCAAA | 55 | 284 |
|  | 2^st^ | MCRT_F2_F5 | Forward | TTTTCATCACTTTTTGCTCCTTT |  |  |
|  |  | MCRT_F2_R7 | Reverse | GCAATGGAAACTATTCCCTTACA | 55 | 227 |
|  | 2^st^ | MCRT_F2_F3 | Forward | GGAATAGTTTCCATTGCATACG |  |  |
|  |  | MCRT_F2_R5 | Reverse | GCAAGTAGTAAGGTGCATA | 51 | 575 |
| F3 | 1^st^ | MCRT_F3_F1 | Forward | CTGAAATTGGCTCAAACATT |  |  |
|  |  | MCRT_F3_R1 | Reverse | TGGTTCCCTAACAACATCACC | 49 | 1030 |
|  | 2^st^ | MCRT_F3_F1 | Forward | CTGAAATTGGCTCAAACATT |  |  |
|  |  | MCRT_F3_R3 | Reverse | GGAAGGGAAGATGTTTTGGA | 49 | 484 |
|  | 2^st^ | MCRT_F3_F2 | Forward | GCGGTCTTGGTATGGCTAAA |  |  |
|  |  | MCRT_F3_R1 | Reverse | TGGTTCCCTAACAACATCACC | 55 | 661 |
|  | 2^st^ | MCRT_F2_F4 | Forward | TATGGGGCATTTTTCTTTGC |  |  |
|  |  | MCRT_F3_R1 | Reverse | TGGTTCCCTAACAACATCACC | 55 | 405 |
| F4 | 1^st^ | MCRT_F4_F1 | Forward | CCAAGGACCAGCAATAGCAA |  |  |
|  |  | MCRT_F4_R1 | Reverse | AAGTATCGGCAAATTAAAAACG | 50 | 1276 |
|  | 2^st^ | MCRT_F4_F1 | Forward | CCAAGGACCAGCAATAGCAA |  |  |
|  |  | MCRT_F4_R3 | Reverse | GCACACGGTATAGGTTTAAGCA | 55 | 571 |
|  | 2^st^ | MCRT_F4_F2 | Forward | GGTTGGCTATCTCATTGGTTC |  |  |
|  |  | MCRT_F4_R1 | Reverse | AAGTATCGGCAAATTAAAAACG | 50 | 860 |
