# Supplementary material for: Genetic analysis of the orthologous crt and mdr1 genes in Plasmodium malariae from Thailand and Myanmar
Source: Malar J. 2020 Aug 31;19:315. doi: 10.1186/s12936-020-03391-6 (PMC7461347; doi:10.1186/s12936-020-03391-6)
Supplement: Supplementary file 2 — Additional file 2. The PCR primers and condition for amplification of pmmdr1 gene. [file 12936_2020_3391_MOESM2_ESM.docx]

**Additional file 2.** The PCR primers and condition for amplification of *pmmdr1* gene

| **Fragment** | **Nested PCR** | **Name** | **Direction** | **Sequence (5'-3')** | **Annealing temperature (^o^C)** | **PCR product (bp)** |
| --- | --- | --- | --- | --- | --- | --- |
| F1 | 1^st^ | MMDR1_F98 | Forward | CCGACATAGCAGCAAACAGA | **57** | 1692 |
|  |  | MMDR1_R1400 | Reverse | GGTCATTATCCCCGTTCTGA |  |  |
|  | 2^st^ | MMDR1_F98 | Forward | CCGACATAGCAGCAAACAGA | **57** | 1036 |
|  |  | MMDR1_R900 | Reverse | TAATGCTCTGTTTACGCATGTC |  |  |
|  | 2^st^ | MMDR1_F120 | Forward | GGGACTTTGGAGTTGTTTAGG | **57** | 1408 |
|  |  | MMDR1_R1400 | Reverse | GGTCATTATCCCCGTTCTGA |  |  |
| F2 | 1^st^ | MMDR1_F1100 | Forward | GTGGTAATACAGGTGATGTCT | **53** | 1809 |
|  |  | MMDR1_R2900 | Reverse | GCAAACACACGCATCAAAAT |  |  |
|  | 2^st^ | MMDR1_F1100 | Forward | GTGGTAATACAGGTGATGTCT | **55** | 1130 |
|  |  | MMDR1_R2100 | Reverse | TTTCATTGTCGTTTCCCACA |  |  |
|  | 2^st^ | MMDR1_F2000 | Forward | CAGATCTAACAAGGGCAGTGA | **55** | 764 |
|  |  | MMDR1_R2900 | Reverse | GCAAACACACGCATCAAAAT |  |  |
| F3 | 1^st^ | MMDR1_F2500 | Forward | ATGCACCAGGTTTGTTATTA | **53** | 1976 |
|  |  | MMDR1_R4884 | Reverse | ACTTCCCTTGCCCTCTCATT |  |  |
|  | 2^st^ | MMDR1_F2500 | Forward | ATGCACCAGGTTTGTTATTA | **51** | 1045 |
|  |  | MMDR1_R3700 | Reverse | CGTTCTTTCGAATGTTTGTT |  |  |
|  | 2^st^ | MMDR1_F3600 | Forward | TGGAAGGAGGAGGAATACCC | **57** | 883 |
|  |  | MMDR1_R4884 | Reverse | ACTTCCCTTGCCCTCTCATT |  |  |
