# Supplementary material for: Genetic analysis of the orthologous crt and mdr1 genes in Plasmodium malariae from Thailand and Myanmar
Source: Malar J. 2020 Aug 31;19:315. doi: 10.1186/s12936-020-03391-6 (PMC7461347; doi:10.1186/s12936-020-03391-6)
Supplement: Supplementary file 3 — Additional file 3. The equivalence position of pmmdr1 gene compared with pfmdr1 and pvmdr1. [file 12936_2020_3391_MOESM3_ESM.docx]

**Additional file 3.** The equivalence position of *pmmdr1* gene compared with *pfmdr1* and *pvmdr1*

| ***Pfmdr1*** | | ***Pvmdr1*** | | ***Pmmdr1*** | | | |
| --- | --- | --- | --- | --- | --- | --- | --- |
| **Amino acids** | **Reference** | **Amino acids** | **Reference** | **Amino acids** | **Nucleotide position** | **Total (N=95)**  **%** | **Reference** |
| K6 | - | R6 | - | **N6I** | **17 (AAC>ATC)** | 2.10 | This study |
| E7 | - | Q7 | - | **Y7C** | **20 (TAT>TGT)** | 5.2 | This study |
| N37 | - | T42T | Kittichai et al., 2018 | S38 | **-** | - | - |
| K39 | - | K44K | Kittichai et al., 2018 | N40 | **-** | - | - |
| S76 | - | S81 | - | **S77** | **231 (TCC>TCT)** | 9.4 | This study |
| N86Y | Thaita et al., 2018 | N91 | - | N87 | **-** | - | - |
| K128 | - | K133N | Imwong et al., 2008 | K129 | - | - | - |
| S134 | - | S139R | Imwong et al., 2008 | S135 | - | - | - |
| G167 | - | G172G | Kittichai et al., 2018 | G168 | - | - | - |
| Y184F | Thaita et al., 2018 | Y189 | - | Y185 | - | - | - |
| N307 | - | N312N | Kittichai et al., 2018 | K308 | - | - | - |
| T350 | - | T355T | Kittichai et al., 2018 | T351 | - | - | - |
| K451 | - | K456I | Kittichai et al., 2018 | K453 | - | - | - |
| S461 | - | S466 | - | **S463** | **1389 (AGC>AGT)** | 42.10 | This study |
| L465 | - | L470H | Kittichai et al., 2018 | L467 | - | - | - |
| M488 | - | L493L | Kittichai et al., 2018 | **L490I** | **1468 (CTT>ATT)** | 4.2 | This study |
| - | - | S513R | Kittichai et al., 2018 | K510 | - | - | - |
| M512 | - | T529T | Kittichai et al., 2018 | N527 | - | - | - |
| L600 | - | L617 | - | **L615** | **1845 (TTG>TTA)** | 31.5 | This study |
| N655 | - | K672N | Kittichai et al., 2018 | D670 | - | - | - |
| N706 | - | N740D | Kittichai et al., 2018 | D722 | - | - | - |
| T722 | - | R756R | Kittichai et al., 2018 | V738 | - | - | - |
| M729 | - | A763V | Kittichai et al., 2018 | I745 | - | - | - |
| P772 | - | P808P | Kittichai et al., 2018 | P794 | - | - | - |
| L810 | - | L845F | Kittichai et al., 2018 | L831 | - | - | - |
| Y825 | - | A861E | Kittichai et al., 2018 | K847 | - | - | - |
| L872 | - | M908L | Kittichai et al., 2018 | L894 | - | - | - |
| S877 | - | S913S | Kittichai et al., 2018 | S899 | - | - | - |
| L900 | - | L936F | Kittichai et al., 2018 | L922 | - | - | - |
| M922 | - | T958M | Kittichai et al., 2018 | M944 | - | - | - |
| Y940 | - | Y976F | Imwong et al., 2008, Kittichai et al., 2018 | Y962 | - | - | - |
| N943 | - | F979S | Kittichai et al., 2018 | L965 | - | - | - |
| M944 | - | M980V | Kittichai et al., 2018 | M966 | - | - | - |
| E960 | - | E996Q | Kittichai et al., 2018 | E982 | - | - | - |
| S1034C | Thaita et al., 2018 | S1071 | - | S1057 | - | - | - |
| L1039 | - | F1076L | Imwong et al., 2008, Kittichai et al., 2018 | **L1063F** | **3189 (TTA>TTC)** | 16.8 | This study |
| N1042D | Thaita et al., 2018 | N1079 | - | N1066 | - | - | - |
| Q1216 | - | K1261E | Imwong et al., 2008 | H1250 | - | - | - |
| M1101 | - | I1138I | Kittichai et al., 2018 | I1125 | - | - | - |
| P1139 | - | P1177T | Kittichai et al., 2018 | P1164 | - | - | - |
| N1193 | - | G1232C | Kittichai et al., 2018 | K1218 | - | - | - |
| S1214 | - | S1257 | - | **N1248I** | **3743 (AAC>ATC)** | 5.26 | This study |
| Q1216 | - | K1261E | - | H1250 | - | - | - |
| A1220 | - | G1265W | Kittichai et al., 2018 | N1262 | - | - | - |
| T1224 | - | T1269 | - | **T1266S** | **3796 (ACA>TCA)** | 12.63 | This study |
| N1229 | - | S1274R | Kittichai et al., 2018 | S1271 | - | - | - |
| D1246Y | Bai et al., 2018 | D1291 | - | D1288 | - | - | - |
| S1313 | - | S1358S | Kittichai et al., 2018 | S1355 | - | - | - |
| R1319 | - | R1364 | - | **R1361S** | **4083 (AGA>AGT)** | 3.15 | This study |
| K1348 | - | K1393N | Kittichai et al., 2018 | K1394 | - | - | - |
| E1351 | - | E1396E | Kittichai et al., 2018 | E1393 | - | - | - |
| T1364 | - | T1409 | - | **T1406S** | **4216 (ACT>TCT)** | 4.2 | This study |
| A1418 | - | A1463 | - | **A1460S/ A1460T** | **4378**  **(GCC>T/ACC)** | 4.2/ 2.10 | This study |
