# Supplementary material for: Genetic analysis of the orthologous crt and mdr1 genes in Plasmodium malariae from Thailand and Myanmar
Source: Malar J. 2020 Aug 31;19:315. doi: 10.1186/s12936-020-03391-6 (PMC7461347; doi:10.1186/s12936-020-03391-6)
Supplement: Supplementary file 4 — Additional file 4. Summary of point mutations in pmcrt and pmmdr1 and mixed P. malariae infections. [file 12936_2020_3391_MOESM4_ESM.docx]

**Additional file 4.** Summary of point mutations in *pmcrt* and *pmmdr1* and mixed *P. malariae* infections

| Gene | Haplotype* (wild type/mutations) | Thailand | | Myanmar | |
| --- | --- | --- | --- | --- | --- |
|  |  | Mono-infection  % (N) | Mixed infection  % (N) | Mono-infection  % (N) | Mixed infection  % (N) |
| *pmcrt* | 4 (wild type) | 87.71 (6) | 14.29 (1) | 76.67 (23) | 23.33 (7) |
|  | 1-3 (mutations) | 77.27 (34) | 22.72 (10) | 78.57 (11) | 21.48 (3) |
| *pmmdr1* | 16 (wild type) | 80 (24) | 26.67 (8) | 76 (19) | 24 (6) |
|  | 1-15 (mutations) | 85.71 (18) | 14.29 (3) | 78.95 (15) | 21.05 (4) |

*Haplotype is referred to haplotype patterns in Table 3 and 5
