# Supplementary material for: Genetic analysis of the orthologous crt and mdr1 genes in Plasmodium malariae from Thailand and Myanmar
Source: Malar J. 2020 Aug 31;19:315. doi: 10.1186/s12936-020-03391-6 (PMC7461347; doi:10.1186/s12936-020-03391-6)
Supplement: Supplementary file 5 — Additional file 5. Summary of point mutations in pmcrt and pmmdr1 and sampling times. [file 12936_2020_3391_MOESM5_ESM.docx]

**Additional file 5.** Summary of point mutations in *pmcrt* and *pmmdr1* and sampling times

| Gene | Haplotype* (wild type/mutations) | Thailand | | Myanmar |
| --- | --- | --- | --- | --- |
|  |  | Year  2002-2008  % (N) | Year  2012-2016  % (N) | Year 2009  % (N) |
| *pmcrt* | 4 (wild type) | 18.52 (5) | 8.33 (2) | 68.18 (30) |
|  | 1-3 (mutations) | 81.48 (22) | 91.67 (22) | 31.82 (14) |
| *pmmdr1* | 16 (wild type) | 55.56 (15) | 62.50 (15) | 56.82 (25) |
|  | 1-15 (mutations) | 44.44 (12) | 37.50 (9) | 43.18 (19) |

*Haplotype is referred to haplotype patterns in Table 3 and 5
